# The Every Woman Every Child initiative: supporting countries in Latin America and the Caribbean to reduce social inequalities in health

**DOI:** 10.1186/s12939-022-01682-9

**Published:** 2022-06-14

**Authors:** Antonio Sanhueza, Liliana Carvajal, Daniel A. Cueva, Sonja Caffe, Alma Virginia Camacho, María Alejandra Berroterán, Deborah Horowitz, Amparo Gordillo-Tobar, Oscar J. Mujica, Every Woman Every Child Latin America and the Caribbean

**Affiliations:** 1 Department of Evidence and Intelligence for Action in Health, Pan American Health Organization, PAHO/WHO, Washington, DC USA; 2 Division of Data Analytics Planning and Monitoring, Data and Analytics Section, UNICEF, NY New York, USA; 3 Department of Global Public Health, Karolinska Institutet, Stockholm, Sweden; 4 Independent Consultant, Santiago, Chile; 5 Family, Promotion and Life Course Department, Pan American Health Organization, PAHO/WHO, Washington, DC USA; 6 United Nations Population Fund, Regional Office for Latin American and the Caribbean (UNFPA LACRO), Panama City, Panama; 7 Communications Officer, Regional Office for Latin American and the Caribbean, UNICEF, Panama City, Panama; 8 Bureau for Latin America and the Caribbean, United States Agency for International Development, Washington, DC USA; 9 Health Nutrition and Population, World Bank, Washington, DC USA

**Keywords:** Women's, Children's and Adolescents' Health, Social inequalities in health, Latin America and the Caribbean, Sustainable Development Goals, Salud de mujeres, niños, niñas y adolescentes, Desigualdades sociales en salud, América Latina y el Caribe, Objetivos de Desarrollo Sostenible

## Abstract

The Every Woman Every Child Latin America and the Caribbean (EWEC-LAC) initiative was established in 2017 as a regional inter-agency mechanism. EWEC-LAC coordinates the regional implementation of the *Global Strategy for Women’s, Children’s and Adolescents’ Health* in Latin America and the Caribbean (LAC), including adaptation to region specific needs, to end preventable deaths, ensure health and well-being and expand enabling environments for the health and well-being of women, children and adolescents. To advance the equitable achievement of these objectives, EWEC-LAC’s three working groups collectively support LAC countries in measuring and monitoring social inequalities in health, advocating for their reduction, and designing and implementing equity-oriented strategies, policies and interventions. This support for data-driven advocacy, capacity building, and policy and program solutions toward closing current gaps ensures that no one is left behind. Members of EWEC-LAC include PAHO, UNAIDS, UNFPA, UNICEF, UN WOMEN, the World Bank, the Inter-American Development Bank, USAID, LAC Regional Neonatal Alliance, and the LAC Regional Task Force for the Reduction of Maternal Mortality. To date, EWEC-LAC has developed and collected innovative tools and resources and begun to engage with countries to utilize them to reduce equity gaps. These resources include a framework for the measurement of social inequalities in health, data use and advocacy tools including a data dashboard to visualize trends in social inequalities in health in LAC countries, a methodology for setting targets for the reduction of inequalities, and a compendium of tools, instruments and methods to identify and address social inequalities in health. EWEC-LAC has also engaged regionally to emphasize the importance of recognizing these inequalities at social and political levels, and advocated for the reduction of these gaps. Attention to closing health equity gaps is ever more critical in the face of the COVID-19 pandemic which has exploited existing vulnerabilities. More equitable health systems will be better prepared to confront future health shocks.

## Resumen

La iniciativa Todas las mujeres, Todos los niños América Latina y el Caribe (EWEC-LAC, por su sigla en inglés) se estableció en 2017 como un mecanismo interinstitucional regional. Coordina la implementación regional de la *Estrategia Mundial para la Salud de la Mujer, el Niño y el Adolescente* en América Latina y el Caribe (ALC), incluyendo la adaptación a necesidades específicas de la región, para poner fin a muertes evitables, garantizar la salud y el bienestar y ampliar entornos propicios para la salud y el bienestar de mujeres, niños, niñas y adolescentes. Para promover el logro equitativo de estos objetivos, los tres grupos de trabajo de EWEC-LAC colectivamente apoyan a los países de ALC en la medición y monitoreo de las desigualdades sociales en salud, la abogacía por la disminución de estas y el diseño e implementación de estrategias, políticas e intervenciones orientadas a la equidad. Este apoyo para cerrar brechas actuales asegura que nadie se quede atrás. Miembros de EWEC-LAC incluyen FPNU, OPS, ONU Mujeres, ONUSIDA, UNICEF, el Banco Mundial, el Banco Interamericano de Desarrollo, USAID, la Alianza Regional Neonatal para ALC, y el Grupo de Trabajo Regional para la Reducción de la Mortalidad Materna. A la fecha, EWEC-LAC ha desarrollado y recopilado herramientas y recursos innovadores y ha comenzado a colaborar con los países para utilizarlos a fin de reducir brechas de equidad. Estos incluyen un marco de medición de desigualdades sociales en salud, herramientas de promoción de datos incluyendo un tablero de datos para visualizar tendencias en desigualdades sociales en salud, una metodología para establecer metas en la disminución de las desigualdades y un compendio de herramientas y métodos para identificar y abordar las desigualdades sociales en salud. EWEC-LAC ha trabajado en la región para enfatizar la importancia de reconocer estas desigualdades a niveles sociales y políticos, y ha abogado por la disminución de éstas. La atención para cerrar las brechas de equidad en salud es cada vez más crítica frente a la pandemia de COVID-19, que ha agudizado las vulnerabilidades existentes. Sistemas de salud más equitativos estarán mejor preparados para hacer frente a futuras crisis de salud.

## Background

In 2013, the Millennium Development Goals (MDGs) were approaching their target year. Despite progress in health in Latin America and the Caribbean (LAC) countries during the MDGs era, differences in health coverage and outcomes among social groups, which we refer to here as social inequalities in health, remained widespread both between and within LAC countries. In this context, the A Promise Renewed for the Americas (APR-LAC) initiative was established to promote cooperation with an equity lens to countries in the Americas and to assist in measuring and monitoring inequalities in reproductive, maternal, children and adolescent health. In 2015, the 2030 Global Agenda for Sustainable Development was launched and the MDGs were superseded by the Sustainable Development Goals (SDGs). Subsequently, the *Global Strategy for Women’s, Children’s and Adolescents’ Health* [1] (*Global Strategy*) was launched in 2016 to “[provide] guidance to accelerate momentum for women’s, children’s and adolescents’ health” and its Operational Framework was approved in the 69^th^ World Health Assembly. The interagency mechanism Every Woman Every Child (EWEC) was created in 2010 by former UN Secretary-General H.E. Ban Ki-moon to catalyze a political movement to advance the health and well-being of women, children and adolescents everywhere and as a framework to support the implementation of the *Global Strategy* on a global level. In 2017, APR-LAC became the regional inter-agency mechanism Every Woman Every Child Latin America and the Caribbean (EWEC-LAC) with a mandate to adapt and implement the *Global Strategy* in the LAC context. Given widespread health inequalities for women, children and adolescents in the LAC region [2], the work of EWEC LAC is critical and also contributes to existing structures that have been ratified by LAC governments such as the *Plan of Action for Women's, Children's, and Adolescents' Health* [3] and the *Roadmap for the Digital Transformation of the Health Sector in the Region of the Americas* [4]. This article provides an overview of the work done by EWEC-LAC to support LAC countries in strengthening their efforts to reduce social inequalities in health.

EWEC-LAC is formed by 8 organizations, namely: the Inter-American Development Bank (IDB), the Pan American Health Organization (PAHO), the Joint United Nations Programme on HIV and AIDS (UNAIDS), the United Nations Population Fund (UNFPA), the United Nations Children's Fund (UNICEF), the United Nations Entity for Gender Equality and the Empowerment of Women (UN WOMEN), the United States Agency for International Development (USAID), and the World Bank (WB). Among some of its collaborative partners are the Regional Task Force for the Reduction of Maternal Mortality (GTR, for its acronym in Spanish) and the LAC Regional Neonatal Alliance. EWEC-LAC has three key areas of work: (i) supporting and promoting the measurement and monitoring of social inequalities in health affecting women, children, and adolescents; (ii) addressing social inequalities in health through evidence-based policy-making, and (iii) advocating for public and political agendas that strive for social equity in the health of women, children, and adolescents. EWEC-LAC is therefore structured in three working groups: (i) the Metrics and Monitoring Working Group (MMWG), (ii) the equity-based Policies, Strategies, and Interventions Working Group (PSIWG), and (iii) the Communications and Advocacy Working Group (CAWG), which work together to advance the initiative’s priorities as detailed below.

### Tracking social inequalities in health

“Health inequities are the unjust differences in health between persons of different social groups, and can be linked to forms of disadvantage such as poverty, discrimination and lack of access to services or goods” [5]. As a normative concept that involves a perception of fairness and justice, health inequity is difficult to measure and track. Hence, health inequalities, which are observable and measurable differences in health between persons of different social groups are used as an indirect means to evaluate health inequity [5]. Their measurement involves a triangulation of two types of information: health-related and social-related. The latter, also known as the equity stratifier, serves to define a social gradient (and hence social groups according to their relative position in that gradient) across which the distribution of the former (either a health coverage or a health outcome indicator) is measured and summarized [5].

To measure social inequalities in the health of women, children, and adolescents in the LAC region, EWEC-LAC has defined a Regional Monitoring Framework that includes 32 priority indicators and 6 equity stratifiers [6]. These indicators were selected in a consultation process with regional experts and national representatives of LAC countries. The Operational Framework of the *Global Strategy* was used as a guide, and emphasis was placed on adapting it to reflect the needs and priorities of the LAC region. The resulting list contains indicators from the SDGs and the *Global Strategy*. Furthermore, it is structured according to the *Global Strategy* Framework that includes Survive, Thrive, and Transform objectives as key pillars encompassing preventable deaths, health and well-being, and enabling environments.

To summarize social inequalities in the health of women, children, and adolescents, EWEC-LAC uses specific methodologies and simple and complex standard inequality measures [5, 7, 8]. The absolute and relative gaps are simple metrics which measure absolute and relative differences, respectively, in health outcomes and coverage between the socially worst-off and better-off groups within a country as defined by the equity stratifier. The Slope Index of Inequality (SII) and the Health Concentration Index (CIX) are two complex metrics that measure absolute and relative gradients, respectively, in health outcomes and coverage across the whole social gradient defined by the equity stratifier.

**Fig. 1:**
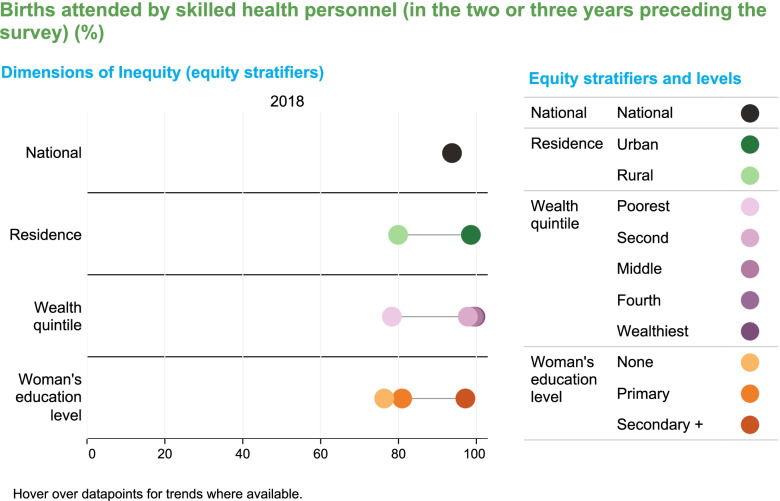
Inequalities in skilled birth attendance in Peru by residence, wealth, and woman’s education

**Fig. 2:**
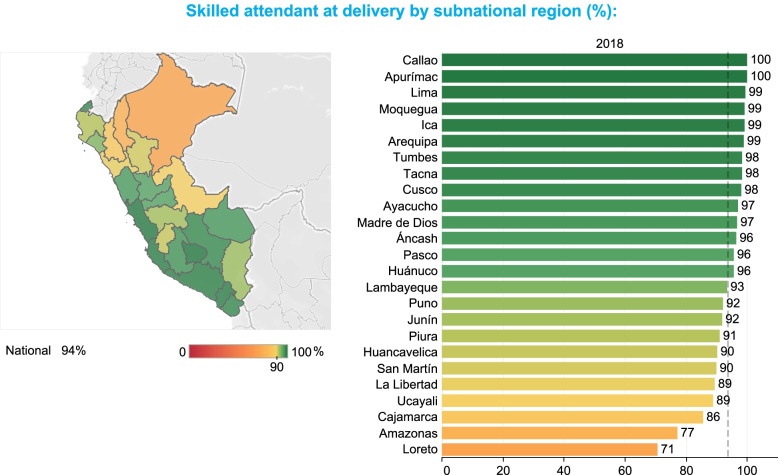
Inequalities in skilled birth attendance in Peru by subnational region

Given that the emphasis of EWEC-LAC’s data work are equity analyses, data for priority indicators predominantly comes from nationally representative survey programs at the household level, such as the Demographic and Health Surveys (DHS) and Multiple Indicator Cluster Survey (MICS) programs, which are a good source for data disaggregated into various socioeconomic characteristics. For some indicators, data may also come from administrative data sources [9, 10]. These data are regularly presented in EWEC-LAC’s data dashboard [11], a tool designed to monitor social inequalities across the prioritized indicators in the EWEC-LAC Regional Monitoring framework as detailed in [6]. Figures 1 and 2 include examples of the presentation of data to track progress. They illustrate absolute inequalities in the coverage of births attended by skilled health personnel in Peru. For instance, in 2018 there were absolute gaps of around 20 percentage points of coverage by residence (households living in rural areas compared to urban areas), by wealth (households in the second, middle, fourth and wealthiest wealth quintiles compared to those in the poorest wealth quintile); and by a woman’s education level (women with no educational attainment compared with those with at least secondary education) (Fig. 1). Furthermore, there are also significant subnational inequalities by geographic areas, with the coverage of births attended by skilled health personnel in Callao exceeding that of Loreto by around 30 percentage points (Fig. 2).

**Fig. 3:**
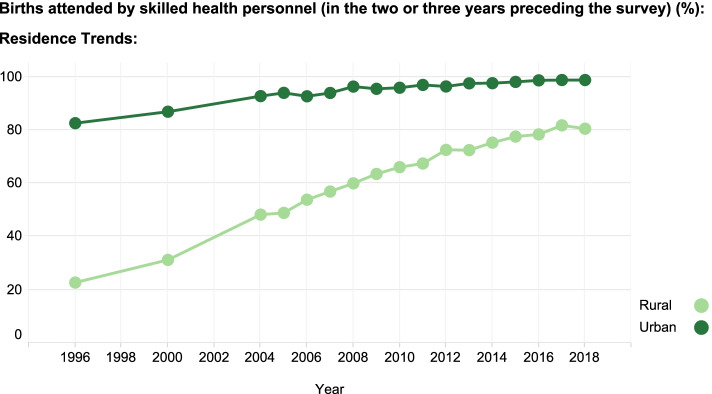
The evolution of skilled birth attendance in rural and urban areas in Peru over time

EWEC-LAC’s data dashboard also presents visualizations based on historical data to track trends. For example, Fig. 3 illustrates that the coverage of births attended by skilled health personnel in rural and urban areas in Peru has increased throughout the years, and the absolute gap in coverage for rural areas compared urban areas has narrowed over time from approximately a 60 percentage point difference in 1996 to around a 20 percentage point difference in 2018. This information is valuable to national and regional policy-makers, as it enables to evaluate trends in health coverage and outcomes and their inequalities, promotes evidence-based policy-making, and allows for equity-targeted efforts and more efficient actions. In the context of the SDG and *Global Strategy* targets set for 2030, the monitoring of health inequalities is useful to track and accelerate their progress, and to create accountability on the promise to leave no one behind.

Another key aspect that promotes reducing social inequalities in health is establishing clear and specific targets for the reduction of current gaps. In this area of work, EWEC-LAC has proposed a methodology to establish SDG 3 (Ensure healthy lives and promote well-being for all at all ages) targets and reduce their inequalities [12]. It consists of an algorithm that allows to set national targets for both the overall progress of SDG 3 indicators and the reduction of inequalities at the subnational level.

### Supporting equity-oriented health actions

EWEC-LAC works with countries in the LAC region to promote the implementation of evidence-based multi-sectoral policies, strategies, and interventions that reduce inequalities in the health of women, children and adolescents and are in line with country priorities within the context of the SDGs and the *Global Strategy*. This work is multi-pronged and involves improving the capabilities of technical country teams to apply equity-based tools and methods; analyzing, documenting and disseminating good practices and lessons learned regarding equity-based approaches; and promoting equity-based policy-dialogue on the health of women, children and adolescents in LAC countries. Collectively, and in combination with efforts to collect and analyze disaggregated data with an equity focus as well as implementation research activities, these actions contribute to improving LAC country capabilities to implement and scale-up evidence-based interventions to reduce national and local inequities in the health of women, children and adolescents.

To support countries in their equity-oriented health actions, EWEC-LAC completed a systematic review of equity-based approaches to address maternal mortality and adolescent pregnancy. Despite progress in some countries, there is a persistent need across the region for strengthening of multisectoral interventions targeting the most vulnerable populations and addressing structural social determinants in health affecting maternal mortality and adolescent pregnancy.

To complement the above resource, EWEC-LAC also developed a compendium of tools, instruments and methods to identify and address social inequities affecting the health and well-being of women, children and adolescents in LAC. The compendium was developed through a systematic search using specific criteria defined by inclusion and exclusion criteria. The search for these tools, instruments and methods was restricted to those published after 2000 in English, Spanish or Portuguese; focused on women, children and adolescents or the general population; and addressing social inequalities in health. Subsequently, the studies that conformed to the criteria were screened and selected independently by two reviewers based on their credibility, dependability, confirmability and transferability; with disagreements resolved by joint review. The compendium is targeted to a wide range of stakeholders, including policy-makers, non-governmental organizations, and academia. It includes key information for each tool, instrument, and method, such as their author, year of design, goals, objectives, design specification, intended users, requirements for implementation, countries and sites where they have been previously implemented, experiences and lessons learned from the implementation, references to access them, and case studies. EWEC-LAC aims to update the compendium periodically, so that it includes state-of-the-art tools, instruments and methods. By providing an overview of these, the compendium may be used to strengthen systematic identification, analysis and responding to social inequities in the health of women, children, and adolescents in LAC.

Another example of key activities conducted by EWEC-LAC was a regional competition to identify good practices applying equity-based approaches in reproductive, maternal, adolescent and child health. To participate, these practices had to respond to a specific health challenge disproportionately affecting women, children or adolescents living in vulnerable situations; incorporate or address intermediary or structural determinants of health and health inequities affecting women, children or adolescents living in vulnerable situations; receive government support to ensure long-term sustainability; be replicable in other contexts and on a larger scale; and have a monitoring and evaluation mechanism to track quantitative or qualitative indicators demonstrating impact or results in relation to practice objectives or goals. The competition resulted in the selection of ten country experiences. The good practices and lessons learned are being documented in a report as well as in video format for sharing in the region for potential utilization in other contexts.

The LAC region represents a disproportionate number of COVID-19 cases and some of the highest mortality rates in the world [2]. In the face of this crisis, EWEC LAC quickly pivoted to advocate for interventions and actions to reduce the direct and indirect effects of the COVID-19 pandemic on women, children and adolescents. EWEC-LAC mobilized a Call to Action and other materials to promote the continuity of essential reproductive, maternal, newborn, child and adolescent services in order to address disruptions in care resulting from mobility restrictions, redirected health resources, and staff shortages. Available data and modeling suggest that there will be significant backtracking in health indicator gains achieved during the last two decades in the region, including a documented excess in maternal deaths due to a combination of primary and secondary impacts [13]. The COVID-19 pandemic has likely exploited existing vulnerabilities to directly and indirectly affect populations already left behind. Thus, support has been provided in close partnership with governments and interagency technical groups to increase understanding of the contributing factors and determinants of excess maternal deaths.

### Advocating for health equity through communication

The implementation of EWEC-LAC’s communication and advocacy activities positions the initiative as a central catalyst for the implementation of the *Global Strategy* in the LAC region. Through these activities, EWEC-LAC identifies key goals, strategies and timelines for the recognition of social inequalities in the health of women, children and adolescents in the LAC region at social and political levels. EWEC-LAC coordinates its communications through social media, newsletters, websites, and public media targeted to specific audiences and tailored to their needs. Furthermore, relevant spokespersons are identified and key messages, taglines and slogans are developed. EWEC-LAC’s communication activities to advocate for health equity also include the creation and dissemination of technical products, which are used in training workshops, and advocacy materials.

In the context of the COVID-19 pandemic, it is essential to prioritize the health of women, children and adolescents in the LAC region. EWEC-LAC developed an infographic providing five methodological recommendations to improve institutional capabilities to monitor inequalities in the health of pregnant women during and after the COVID-19 pandemic [14]. These recommendations included the following components: governments should (1) include indicators related to COVID-19 as part of the basic health indicator framework for pregnant women; (2) systematically and periodically collect data for this framework, ensuring it is regularly updated and included in the health information systems; (3) explore the presence, magnitude and trends of social inequalities in the health of pregnant women; (4) design a data dashboard to visualize maternal health and its inequalities over time; and (5) establish consensual and explicit goals to improve national averages and reduce social inequalities for health indicators for pregnant women. By improving institutional capabilities, EWEC-LAC aspires that cooperation with policy-makers and staff from Ministries of Health from LAC countries may result in improved health for pregnant women regardless of their socioeconomic background.

## Conclusion

EWEC-LAC promotes the implementation of evidence-based strategies to reduce social inequalities in the health of women, children and adolescents across the LAC region. Building on preliminary work of the APR-LAC movement, since 2017, EWEC-LAC has played a key role in the implementation of the *Global Strategy* in a targeted manner that considers contextual realities and addresses the specific challenges faced in the LAC region. The three working groups of EWEC-LAC work together to support LAC countries in measuring and monitoring their social inequalities in health, implementing equity-based public health strategies, policies and interventions, and advocating for the reduction of social inequalities of health in the region.

EWEC-LAC acknowledges current challenges to achieving health equity in the LAC region. Importantly, the COVID-19 pandemic has disrupted the normal functioning of the health system, affected the coverage and quality of health services, and required governments to direct their funding of public health towards policies and actions mitigating the spread of COVID-19. As such, it poses a threat to equity in the health of women, children and adolescents and is exacerbating existing widespread within-country and between-country social inequalities and derailing progress towards the SDGs.

Although the COVID-19 pandemic has affected the availability of recent data, complementing survey data with data from administrative sources allows to continue to monitor health inequalities among LAC countries during the pandemic. Nonetheless, it is essential to further improve the collection, reporting, and monitoring of these data, which would allow for greater room for evidence-based policy-making and advocacy for the reduction of social inequalities in health. Particularly, it is important that relevant stakeholders, such as ministries of health, ministries of finance, national statistical institutes, and public and private academic institutions and research centers, collaborate to strengthen national information systems to ensure the collection and reporting for health indicators for women, children, and adolescents disaggregated by equity stratifiers, with data disaggregated at a minimum by age and other relevant characteristics, such as place of residence (rural or urban), race, ethnicity, occupation, education, or socioeconomic status, as well as subnational geographic regions. This would allow countries to monitor social inequalities in health, and especially the inequalities between subgroups to identify those that are left behind, as in [15], as well as promote intersectoral collaboration, policy formulation, and investment in public health. It is also essential that countries adopt lessons learned and good practices, such as the strengthening of intersectoral work and action at subnational levels, the potential to merge data from different social sectors using the subnational region as a unique common identifier between databases, the corresponding articulation of more holistic interventions that can improve health, and the establishment of health inequality observatories. Furthermore, universities play a key role in training professionals on heath inequality matters by including courses which cover their measurement, monitoring, and the social determinants of health, as a standard part of postgraduate programs in public health and related areas.

The work with technical teams from LAC countries has achieved important results to date, such as improving capabilities in applying methodologies for measuring and monitoring inequalities in health. For instance, central technical teams within South American countries were formed and trained in measuring and monitoring health inequalities. Notably, the central team in Chile subsequently trained teams at the subnational regional level, and each of these subnational teams developed profiles of inequalities within each region of the country [16]. Nonetheless, the institutionalization of the measurement and monitoring of inequalities at the political level and improving the capabilities of country governments to implement policies and approaches that address these identified gaps remain key challenges in this area of work. Furthermore, the translation of the quantitative evidence on social inequalities in health into the articulation and implementation of pro-equity policies and interventions is an additional challenge.

Finally, it should be noted that there are multidimensional factors affecting the health and well-being of women, children and adolescents which underscores the importance of a multisectoral approach in which governmental and non-governmental sectors, academia and civil society work jointly to leverage competitive advantages, increase accountability, and ensure an inclusive approach. As 2030, the target year for the *Global Strategy* and SDGs, approaches, EWEC-LAC remains committed to overcoming these challenges. Now, more than ever, inequity issues could not be left aside. Consequently, for the near term future, EWEC-LAC seeks to promote the reimagining and rebuilding of health systems in the region in the post-COVID-19 era with a focus on equity, and continue supporting countries in strengthening their efforts to promote equity-based policies.

## Data Availability

Not applicable.

## Abbreviations

APR-LAC: A Promise Renewed for the Americas
CAWG: Communications and Advocacy Working Group
DHS: Demographic and Health Surveys
EWEC: Every Woman Every Child
EWEC-LAC: Every Woman Every Child Latin America and the Caribbean
Global Strategy: Global Strategy for Women’s, Children’s and Adolescents’ Health
GTR (acronym in Spanish): Regional Task Force for the Reduction of Maternal Mortality
HCI: Health Concentration Index
IDB: Inter-American Development Bank
LAC: Latin America and the Caribbean
MDGs: Millennium Development Goals
MICS: Multiple Indicator Cluster Survey
MMWG: Metrics and Monitoring Working Group
PAHO: Pan American Health Organization
PSIWG: Equity-based Policies, Strategies and Interventions Working Group
SDGs: Sustainable Development Goals
SII: Slope Index of Inequality
UN WOMEN: United Nations Entity for Gender Equality and the Empowerment of Women
UNAIDS: Joint United Nations Programme on HIV and AIDS
UNFPA: United Nations Population Fund
UNICEF: United Nations Children's Fund
USAID: United States Agency for International Development
